# Biofilm-producing ability of methicillin-resistant *Staphylococcus aureus* clinically isolated in China

**DOI:** 10.1186/s12866-024-03380-8

**Published:** 2024-07-03

**Authors:** Jingyi Yu, Weihua Han, Yanlei Xu, Li Shen, Huilin Zhao, Jiao Zhang, Yanghua Xiao, Yinjuan Guo, Fangyou Yu

**Affiliations:** 1 Department of Clinical Laboratory, Shanghai Pulmonary Hospital, School of Medicine, Tongji University, Shanghai, China; 2 Department of Clinical Laboratory, Key Laboratory of Clinical Laboratory Diagnosis and Translational Research of Zhejiang Province, The First Affiliated Hospital of Wenzhou Medical University, Wenzhou, Zhejiang China; 3 School of Public Health, Nanchang University, Nanchang, Jiangxi China

**Keywords:** Methicillin-resistant *Staphylococcus aureus*, Biofilm, Adhesion genes

## Abstract

**Background:**

*Staphylococcus aureus*, a commensal bacterium, colonizes the skin and mucous membranes of approximately 30% of the human population. Apart from conventional resistance mechanisms, one of the pathogenic features of *S. aureus* is its ability to survive in a biofilm state on both biotic and abiotic surfaces. Due to this characteristic, *S. aureus* is a major cause of human infections, with Methicillin-Resistant *Staphylococcus aureus* (MRSA) being a significant contributor to both community-acquired and hospital-acquired infections.

**Results:**

Analyzing non-repetitive clinical isolates of MRSA collected from seven provinces and cities in China between 2014 and 2020, it was observed that 53.2% of the MRSA isolates exhibited varying degrees of ability to produce biofilm. The biofilm positivity rate was notably high in MRSA isolates from Guangdong, Jiangxi, and Hubei. The predominant MRSA strains collected in this study were of sequence types ST59, ST5, and ST239, with the biofilm-producing capability mainly distributed among moderate and weak biofilm producers within these ST types. Notably, certain sequence types, such as ST88, exhibited a high prevalence of strong biofilm-producing strains. The study found that SCC*mec* IV was the predominant type among biofilm-positive MRSA, followed by SCC*mec* II. Comparing strains with weak and strong biofilm production capabilities, the positive rates of the *sdrD* and *sdrE* were higher in strong biofilm producers. The genetic determinants *ebp*, *icaA*, *icaB*, *icaC*, *icaD*, *icaR*, and *sdrE* were associated with strong biofilm production in MRSA. Additionally, biofilm-negative MRSA isolates showed higher sensitivity rates to cefalotin (94.8%), daptomycin (94.5%), mupirocin (86.5%), teicoplanin (94.5%), fusidic acid (81.0%), and dalbavancin (94.5%) compared to biofilm-positive MRSA isolates. The biofilm positivity rate was consistently above 50% in all collected specimen types.

**Conclusions:**

MRSA strains with biofilm production capability warrant increased vigilance.

**Supplementary Information:**

The online version contains supplementary material available at 10.1186/s12866-024-03380-8.

## Background

*Staphylococcus aureus* is a Gram-positive pathogen that causes various skin infections globally each year, as well as life-threatening invasive infections. It is also a major pathogen in pneumonia and other respiratory infections, prosthetic joints, surgical sites, cardiovascular infections, and hospital-acquired bacteremia [1, 2].

The development and formation of biofilm involve four stages: attachment of planktonic cells to the surface, colonization and biofilm formation, biofilm maturation, and biofilm dispersion [3, 4]. When an implant is introduced into the host, the immune response easily covers its non-biological surface with host proteins [5, 6]. Subsequently, *S. aureus* initiates a cycle of biofilm formation that involves the expression of adhesion matrix molecules (MSCRAMM), including aggregation factors ClfA, ClfB [7], fibronectin-binding proteins FnbA, FnbB, and serine-aspartate repeat proteins SdrC, SdrD, and SdrE [8]. All of these factors facilitate implant surface colonization by binding to the host through tight adherence proteins. Once attached, biofilm proliferation occurs through the secretion of DNA, polysaccharides, and proteins. Ica proteins (IcaADBC) aid in the accumulation of polysaccharide intercellular adhesin (PIA), IcaR suppresses *icaADBC* transcription by binding to a 42 bp sequence in the icaR-*icaA* intergenic region [9]. As proliferation continues within the matrix, cells lose direct contact with the implant surface and host proteins, relying on cell-cell and extracellular polymeric substance (EPS) adhesion [10]. SdrC and FnBPs, for example, can exert this role through self-association [11, 12], and EbpS [13] binds to elastin peptides and elastin, acting as an adhesin that binds to host cell elastin [14]. As the biofilm matures, microcolonies exhibit different growth characteristics and protein expression depending on their location in the biofilm [15]. Ultimately, biofilms disperse, break down, spread, and repeat the process elsewhere.

Bacteria can transition between planktonic (single-cell) and biofilm forms, growing on solid surfaces and embedding in bacterial communities in the extracellular polymeric matrix. Studies indicate that bacteria in biofilms are 1,000 times more resistant to conventional drugs than planktonic bacteria and are more resilient against attacks from the host immune system [16]. Once formed, biofilms are challenging to eliminate, making the treatment of associated infections difficult [17, 18]. The presence of biofilms helps microorganisms resist and minimize the killing effects of antimicrobial drugs and host defenses, promoting the virulence of *S. aureus* [19, 20].

The objective of this study is to assess the biofilm-forming ability of MRSA collected from tertiary hospitals in China and examine the relationship between biofilm formation, antibiotic resistance, relevant adhesion genes, different ST types, and regions.

## Results

### Biofilm formation analysis

Among 663 MRSA isolates, 53.2% exhibited varying degrees of ability to produce biofilm. Among them, 54 MRSA strains (8.1%) were classified as strong biofilm producers; 19.2% of MRSA were moderate biofilm producers, and 25.9% of MRSA were weak biofilm producers. Nearly half (46.8%) of the MRSA strains did not demonstrate biofilm production ability **(**Table 1**)**. Plots of strong, medium, weak and no biofilm in 96-well plates are shown in Supplementary material 1.

**Table 1:** Classification of biofilm formation abilities by Mtp method

Biofilm formation abilities	No. of isolates	Percentage
Strong	54	8.1
Moderate	127	19.2
Weak	172	25.9
None	310	46.8

### Differences in biofilm phenotypes among strains from different regions

**Fig. 1:**
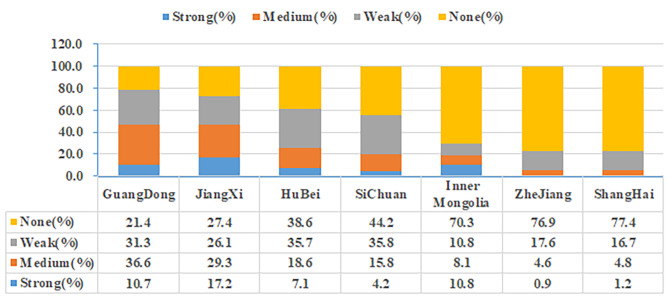
This figure depicts the analysis of biofilm formation capability of MRSA strains from seven provinces and cities in China in the present study

Among the 663 MRSA strains, the regions with biofilm positivity rates exceeding 50% were Guangdong (88/112, 78.6%), Jiangxi (114/157, 72.6%), and Hubei (43/70, 61.4%), followed by Sichuan (53/95, 55.8%). Among the MRSA collected in Guangdong, the proportion of strains strongly positive for biofilm and moderately intense biofilm were 10.7% and 36.6% respectively. The proportion of strains with moderate and weak biofilm formation was relatively high, indicating a balanced biofilm-forming ability in this region. In Jiangxi, 17.2% of strains exhibited strong biofilm positivity, 29.3% were classified as moderate biofilm producers, and 26.1% were weak biofilm producers. The proportions of strains with moderate and weak biofilm formation were relatively high, indicating a balanced biofilm-forming ability in this region. Inner Mongolia, Zhejiang, and Shanghai showed a decreasing trend in biofilm positivity rates, with Inner Mongolia having the highest proportion of strongly positive biofilm strains (Fig. 1). The three cities with the highest rates of MRSA biofilm negativity were Shanghai (65/84, 77.4%), Zhejiang (83/108, 76.9%), and Inner Mongolia (26/37, 70.3%).

### The differences in biofilm phenotypes among different molecular subtypes

In this study, a total of 663 MRSA strains collected from 2016 to 2020 were mainly characterized as moderate and weak biofilm producers, with an overall decreasing trend in the biofilm-forming capability (Fig. 2A). Figure 2B illustrates the biofilm formation abilities of the major sequence types (STs) identified in this study, including ST59, ST5, ST239, ST764. Among these, ST59 exhibited the highest proportions in strong positive, moderately strong, and weak biofilm-producing strains. However, ST764 did not show any strains with strong positive biofilm formation. Other ST Types group includes ST types other than the above 4 ST types.

**Fig. 2A:**
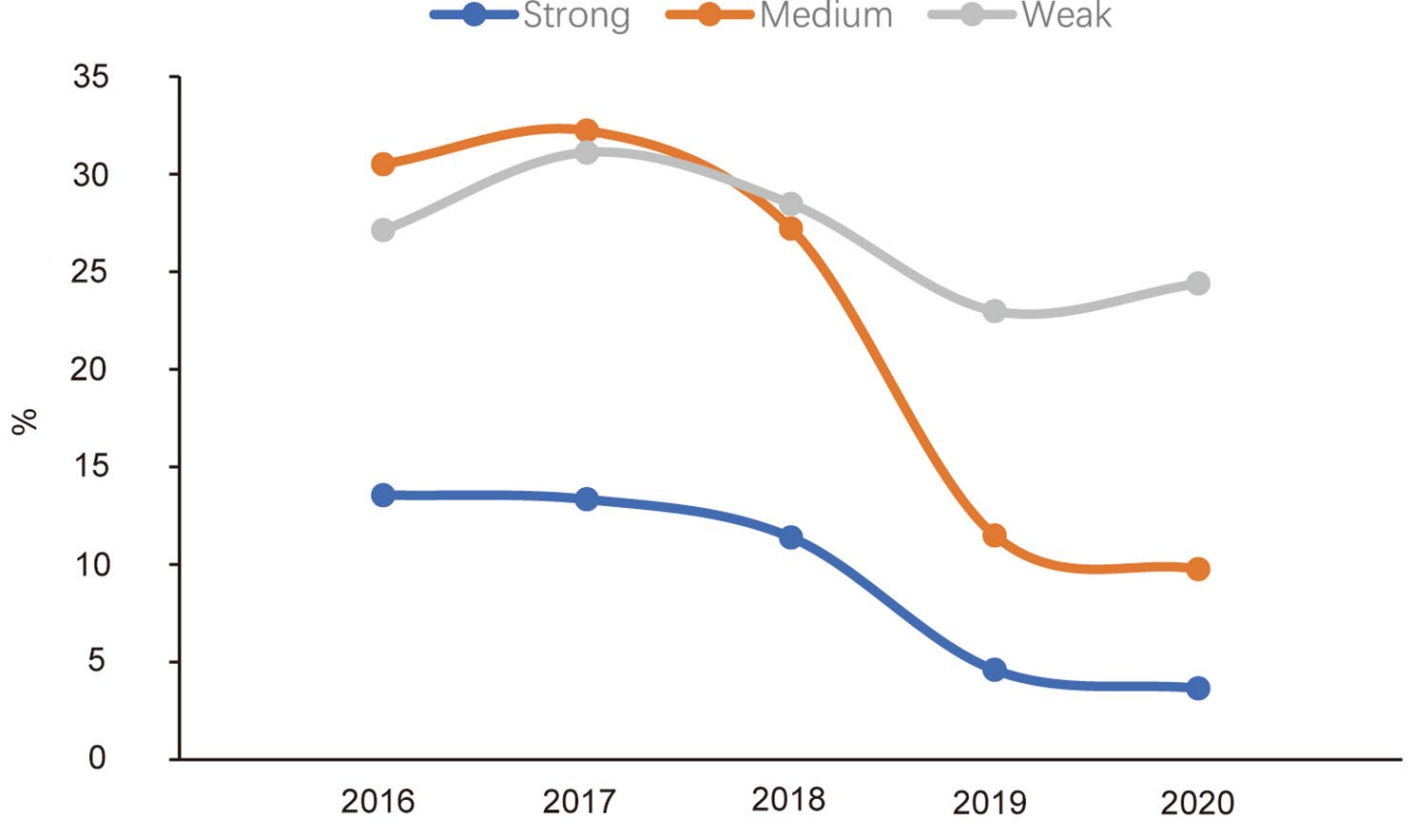
Analysis of MRSA biofilm formation capability collected in this study from 2016 to 2020

**Fig. 2B:**
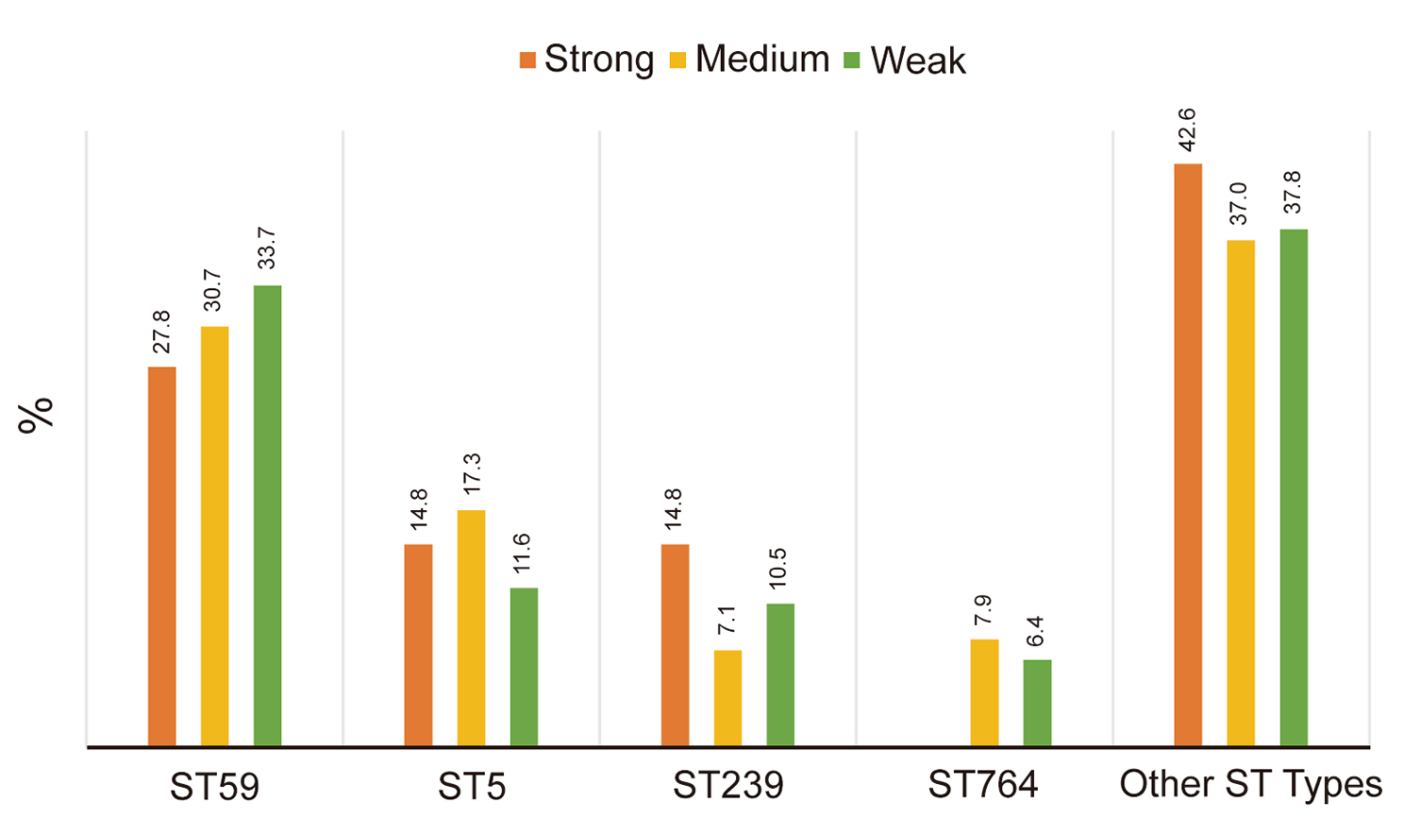
Biofilm formation abilities of major sequence types (STs) in this study

**Fig. 3:**
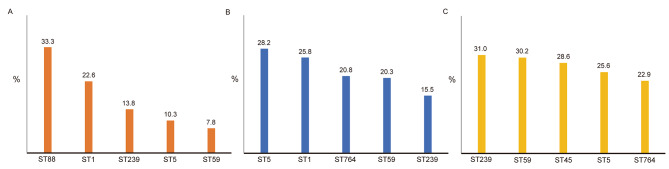
Ratio of biofilm-forming ability. **(A)** Positive rate of major ST types among MRSA with strong biofilm production capacity. **(B)** Positive rates of major ST types among MRSA with moderate biofilm production capacity. **(C)** Positive rate of major ST types in MRSA with weak biofilm production capacity

As shown in Fig. 3, the ST types with higher positive rates of strong biofilms in this study mainly include ST88, ST1, ST239, ST5, and ST59, and the ST types with higher positive rates of moderate biofilms mainly include ST5, ST1, ST764, and ST59, ST239, ST types with higher positive rate of weak biofilm mainly include ST239, ST59, ST45, ST5 and ST764. The biofilm production abilities of ST5, ST59, and ST764 are mainly medium and weak, the biofilm production abilities of ST88 are mainly strong, the biofilm production abilities of ST1 are mainly strong and medium, and the biofilm production abilities of ST45 and ST239 are mainly weak.

**Fig. 4:**
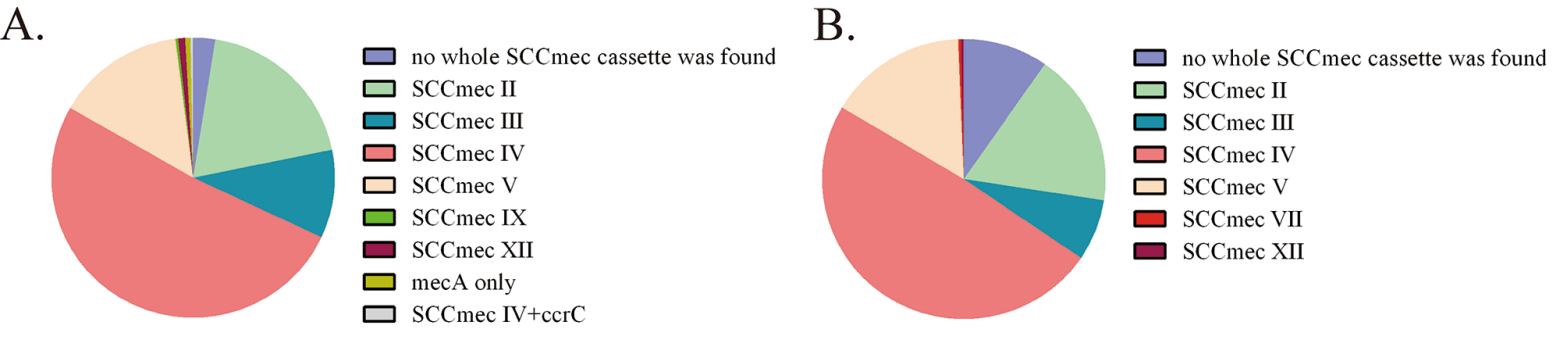
Analysis of biofilm production capabilities in different SCC*mec* types. **(A)** Proportions of various SCC*mec* types in biofilm-positive strains. **(B)** Proportions of various SCC*mec* types in biofilm-negative strains

The results of SCC*mec* typing, as shown in Fig. 4, reveal that biofilm-positive MRSA primarily includes types SCC*mec* IV (181/353, 51.3%), SCC*mec* II (68/353, 19.3%), and SCC*mec* V (52/353, 14.7%). Among these, SCC*mec* IV is the predominant type in biofilm-positive MRSA, followed by SCC*mec* II. Two MRSA strains identified by SCC*mec* Finder as carrying only the *mecA* gene belong to the strong biofilm-positive isolates (2/2, 100.0%). Among biofilm-negative MRSA, SCC*mec* IV (152/310, 49.0%) and SCC*mec* II (55/310, 17.7%) are the most prevalent SCC*mec* types. In the 39 MRSA strains with unknown cassette types, the proportion of biofilm-negative strains is the highest (30/39, 76.9%).

### The differences in biofilm phenotypes among biofilm-related genes

In this study, based on sequencing results analysis, the frequencies of biofilm-related genes *sdrD* and *sdrE* were found to be higher in strong biofilm producers and moderately strong biofilm producers compared to MRSA strains with no biofilm-forming capability (Table 2). We identified several genetic pathways associated with biofilm formation. In strains with strong positive biofilm production, the predominant genes were *ebp*, *icaA*, *icaB*, *icaC*, *icaD*, *icaR*, and *sdrE* (49/54, 90.7%). In moderately strong biofilm-producing MRSA, the main genes were *ebp*, *icaA*, *icaB*, *icaC*, *icaD*, *icaR*, *sdrC*, and *sdrE* (109/127, 85.8%).

**Table 2:** Analysis of the positive rates of relevant Biofilm genes in MRSA with different Biofilm production capacities

Adhesin Gene(s)	Biofilm Formation Abilities				
	Strong(*n* = 54)	Moderate(*n* = 127)	Weak(*n* = 172)	None(*n* = 310)	*p*-value
*aur*	45(83.3%)	114(89.8%)	136(79.1%)	230(74.2%)	**-**
*cap8H*	43(79.6%)	79(62.2%)	111(64.5%)	165(53.2%)	p1:0.0003, p2:0.08612
*cap8I*	43(79.6%)	78(61.4%)	111(64.5%)	166(53.5%)	p1:0.0003, p2:0.1326
*cap8J*	44(81.5%)	79(62.2%)	111(64.5%)	170(54.8%)	p1:0.0002, p2: 0.1579
*cap8K*	43(79.6%)	78(61.4%)	111(64.5%)	166(53.5%)	p1:0.0003, p2:0.1326
*icaA*	54(100%)	125(98.4%)	171(99.4%)	309(99.7%)	**-**
*icaB*	54(100%)	126(99.2%)	171(99.4%)	309(99.7%)	**-**
*icaC*	54(100%)	125(98.4%)	170(98.8%)	308(99.4%)	**-**
*icaD*	54(100%)	126(99.2%)	171(99.4%)	310(100%)	**-**
*icaR*	54(100%)	124(97.6%)	170(98.8%)	310(100%)	**-**
*clfA*	33(61.1%)	95(74.8%)	133(77.3%)	223(71.9%)	**-**
*clfB*	37(68.5%)	92(72.4%)	123(71.5%)	252(81.3%)	**-**
*cna*	12(22.2%)	15(11.8%)	32(18.6%)	79(25.5%)	**-**
*ebp*	54(100%)	120(94.5%)	164(95.3%)	288(92.9%)	**-**
*fnbA*	17(11.6%)	49(38.6%)	54(31.4%)	120(38.7%)	**-**
*fnbB*	7(13%)	11(8.7%)	8(4.7%)	30(9.7%)	**-**
*sdrC*	46(85.2%)	112(88.2%)	141(82%)	244(78.7%)	**-**
*sdrD*	37(68.5%)	71(55.9%)	82(47.7%)	166(53.5%)	p1:0.0409, p2:0.6534
*sdrE*	49(90.7%)	113(89.0%)	145(84.3%)	226(72.9%)	p1:0.0049, p2:0.0003

p1: Statistical analysis between strong biofilm group and none biofilm group;

p2: Statistical analysis between moderate biofilm group and none biofilm group.

### The differences in antimicrobial activity

**Fig. 5:**
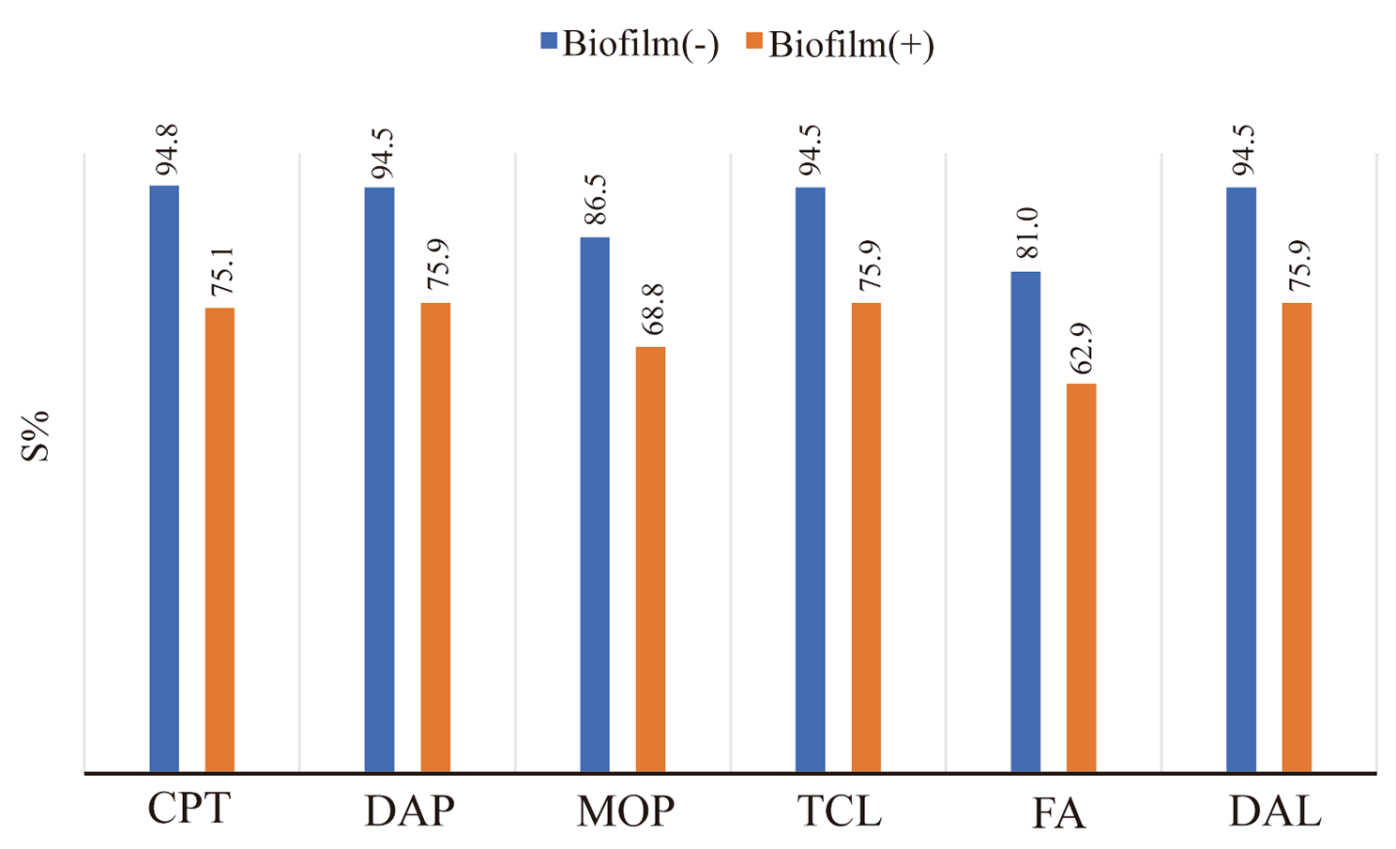
Antimicrobial susceptibility profiles of biofilm-positive and biofilm-negative MRSA isolates

In the 663 MRSA strains, there were 353 biofilm-positive MRSA isolates, and an additional 310 biofilm-negative MRSA isolates. The results of susceptibility testing showed that biofilm-negative MRSA isolates exhibited higher sensitivity rates to Cefalotin (94.8%), Daptomycin (94.5%), Mupirocin (86.5%), Teicoplanin (94.5%), Fusidic acid (81.0%), and Dalbavancin (94.5%) compared to biofilm-positive MRSA isolates (Fig. 5).

### The differences in specimen types

**Fig. 6:**
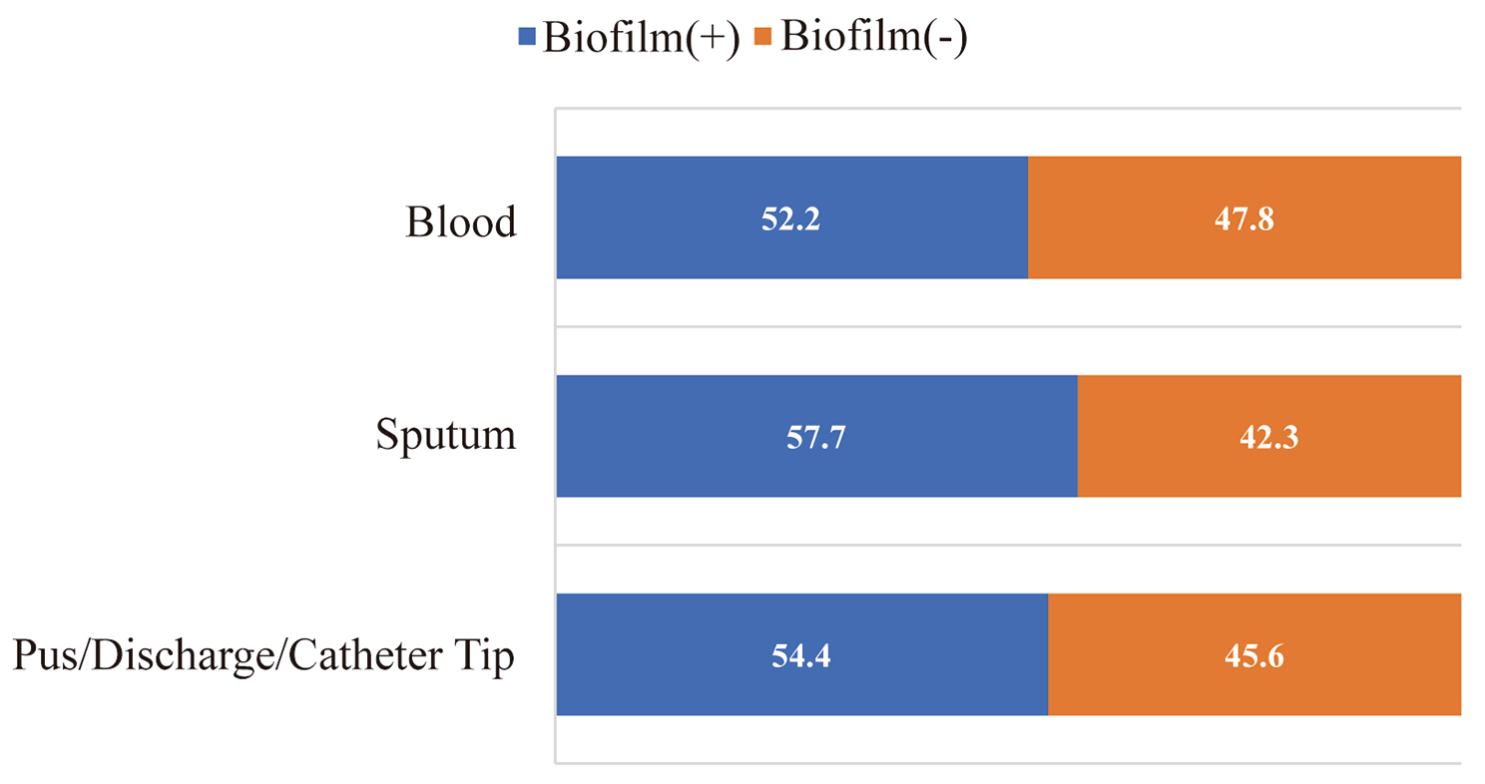
Specimen types of MRSA isolates in this study

In all specimen types, the biofilm positivity rate is consistently above 50%. The positive rate of biofilm among sputum samples was the highest (90/156, 57.7%). Blood specimens have the highest biofilm negativity rate (99/207, 47.8%), followed by pus/ Discharge /catheter tips (119/261, 45.6%) (Fig. 6).

## Discussion

The increasing prevalence of MRSA infections poses a significant threat to human health, and the clinical challenge is particularly pronounced with MRSA strains that possess the ability to produce biofilms. Biofilms, encapsulated in a self-produced extracellular polymeric matrix, adhere to both living and non-living surfaces [21, 22]. The formation of biofilms enhances the pathogenicity and antibiotic resistance of bacteria in adverse environments [23], providing a unique opportunity for sustained infections, antibiotic resistance, and immune evasion [24]. In this study, we sequenced 663 MRSA strains that were prevalent in China over the past six years, assessed their biofilm production capabilities, and analyzed the relationships between biofilm production, different regions in China, various ST types, biofilm-related genes, and antibiotic resistance.

The ability of *S. aureus* to produce biofilm is considered to contribute to issues such as food poisoning, antibiotic resistance, and many other problems [25, 26]. In our survey, 8.1% of MRSA were strong biofilm producers, 19.2% were moderate biofilm producers, and 25.9% were weak biofilm producers. Almost all MRSA strains we collected tested positive for the *icaA*, *icaB*, *icaC*, and *icaR*, and the prevalence of these four genes did not differ in terms of biofilm production capabilities. This suggests that the presence of genes encoding PIA/PNAG is not the sole determinant of biofilm production capability. While Lin Chen et al.‘s study indicated a difference in the detection rate of the *icaD* between strong and weak biofilm-producing strains [27], our results showed that the *icaD* was detected in almost all MRSA strains. Furthermore, compared to isolates without biofilm production capability, isolates with strong biofilm production capability had a higher prevalence of the *sdrD* and *sdrE*. This is partially consistent with previous research [27, 28]. The *cna* is the only recognized *S. aureus* gene encoding a specific collagen-binding adhesin [29]. Our results indicate that among *cna*-positive isolates, those without biofilm production capability had the highest frequency (79/137, 57.7%). Another study reported that *cna*-positive isolates (20%) were identified as moderate or strong biofilm producers [30]. In contrast, Khoramian et al. [31]. found no significant difference in the detection rate of the *cna* between these two groups.

*S. aureus* elastin-binding protein (EbpS) is a complete membrane protein that attaches to host cells by binding to soluble elastin peptides and intact elastin through its exposed N-terminal domain [32]. In this study, the detection rate of the *ebps* in 54 biofilm-strong positive MRSA isolates was 100%, while in biofilm-negative strains, the detection rate of the *ebps* was 92.9% (288/310). Azara and colleagues found that 80.6% of *S. aureus* isolates collected from sheep mastitis samples possessed the *ebps*. Another study indicated that, regardless of their adhesion capabilities, the *ebps* was detected in all MSSA and MRSA clones [33]. The collective findings of these studies suggest that further research is needed to elucidate the role of the *ebps* in the process of biofilm formation.

Barbu EM et al. found that *sdrCDE* knockout strains exhibited a decrease in biofilm formation ability compared to wild-type strains [8]. According to our research results, the percentage of strong biofilm producers carrying *sdrD* was 68.5%, while the detection rate of *sdrD* in biofilm-negative isolates was 53.5%. Similarly, the detection rate of *sdrE* in strong biofilm-producing isolates was 90.7%, in moderately strong biofilm-producing isolates was 89.0%, and in biofilm-negative isolates, the detection rate of *sdrE* was 72.9%. This suggests that SdrD and SdrE may be important molecules for bacterial cell-cell adhesion and subsequent biofilm formation.

Capsular polysaccharide (CP) is a component of bacterial cell walls that promotes cell adhesion to medical devices. *S. aureus* CP has been classified into 11 types, with only types 5 and 8 (encoded by the genes CAP5 and CAP8, respectively) present in 80–90% of clinical strains [34–36]. Studies have found a significant association between capsular genotype and phenotype with the amount of biofilm formation [37]. CP formation is reported to contribute to virulence mechanisms and reduce antibiotic sensitivity [38]. As shown in Table 2, the gene positivity rates for *cap8H*, *cap8I*, *cap8J*, and *cap8K* differ among MRSA strains with different biofilm growth intensities. The gene positivity rates of *cap8H*, *cap8I*, *cap8J*, and *cap8K* in strong biofilm-producing MRSA are higher than in biofilm-negative MRSA, and this difference is statistically significant.

The formation of biofilms contributes to bacterial virulence mechanisms and resistance to antibiotics and harsh environmental conditions. Broadly speaking, there are two mechanisms that lead to biofilm-mediated resistance [39]. The first is due to limited diffusion or exclusion caused by the biofilm matrix itself, preventing antimicrobial drugs from reaching their targets. The second mechanism involves physiological changes in bacteria residing in biofilms compared to planktonic bacteria [40, 41]. In this study, 53.2% of MRSA isolates demonstrated the ability to produce biofilms, and MRSA without biofilm production capability exhibited higher sensitivity to cell wall-targeting antibiotics such as cefalotin, daptomycin, mupirocin, teicoplanin, fusidic acid, and dalbavancin compared to biofilm-producing MRSA.

This study provides crucial information on the biofilm-forming capability of a large-scale collection of MRSA isolates from China for the first time. Among the MRSA strains collected from Guangdong and Jiangxi provinces, the predominant types were moderate and weak biofilm producers. In contrast, in MRSA strains from other provinces, weak biofilm producers were predominant among strains capable of producing biofilms. These regional differences may be associated with various factors such as environmental conditions, host factors, and the genetic background of bacterial strains. These results offer initial insights into the biofilm-forming capabilities of MRSA in different regions. Our study identified a correlation between certain sequence types (STs) and biofilm-forming capability. In this study, ST239 (8/58, 13.8%) biofilm had the highest strong positive rate, followed by ST5 (8/78, 10.3%) and ST59 (15/192, 7.8%).ST5 had the highest overall biofilm positivity rate (50/78, 64.1%). These findings are somewhat consistent with previous research in China [42, 43]. *S. aureus* within biofilms often exhibits reduced responsiveness to antibiotics, significantly limiting the antibiotic choices for clinical treatment of *S. aureus* infections [44]. However, our results indicate that *S. aureus* with strong biofilm-forming capabilities does not consistently demonstrate more severe antibiotic resistance, suggesting the need for further research to explain this phenomenon.

## Conclusions

In conclusion, our study gathered MRSA isolates from multiple tertiary hospitals in China. The comprehensive analysis of biofilm formation, related adhesion genes, antibiotic resistance profiles, regional distribution, and other relevant factors undoubtedly contributes to the control and prevention of MRSA infections in tertiary hospitals.

## Methods

### Sample collection and antimicrobial susceptibility testing

From 2014 to 2020, non-repetitive MRSA isolates were collected from seven provinces and cities in China, including Guangdong, Jiangxi, Hubei, Sichuan, Inner Mongolia, Zhejiang, and Shanghai. In each of these regions, a representative tertiary teaching hospital was selected for in-depth investigation. MRSA isolates were cultured on Columbia blood agar plates at 37 °C (± 1 °C) for 16–18 h. Confirmation was done through colony morphology, Gram staining, cell morphology, catalase, and coagulase tests using standard laboratory procedures. All isolates were re-identified as species using MALDI-TOF MS (Bruker Daltonics GmbH, Bremen, Germany). Before identification, standard calibration mixtures with *Escherichia coli* (ATCC 8739) extracts were used for quality control and calibration. Further antimicrobial susceptibility testing was conducted on all isolates using the standard methods provided by the VITEK 2 Compact system (bioMérieux, Marcy-l’Étoile, France). The strains in this study originated from the research of Wang B et al. [45]and Zhu H et al. [46]. , with the antimicrobial susceptibility test results of MRSA in this study referring to the findings of Wang B and Zhu H.

### Identification of biofilm production ability of MRSA

Based on the previously reported method [47], static biofilm formation assays were conducted using a 96-well polystyrene plate (NEST, Wuxi, China). MRSA obtained from the culture was inoculated at a ratio of 1:100 in TSB medium containing 0.5% glucose, and the cultures were incubated without shaking at 37 °C for 24 h. Additionally, control wells containing only TSBG and MRSA were included. After 24 h of incubation, wells were washed three times with phosphate-buffered saline (PBS). The biofilms were fixed with 99% methanol for 15 min, and excess solution was removed. Subsequently, the biofilms were stained with crystal violet for 10 min and washed with running water until water became colorless. After adding 30% acetic acid, the absorbance at OD_600_ was measured. Following the method of Mohsen Mirzaee et al. [48]. Optical density cut-off (ODc) was determined. It is defined as average OD of negative control + 3×standard deviation (SD) of negative control. Formation of biofilm by isolates was analysed and categorised relying on the absorbance of the crystal violet-stained attached cells. The data calculation has been shown in Table 3. *S. epidermidis* ATCC 35,984 strains were used for strongly biofilm-producing control, while *S. epidermidis* ATCC 12,228 strains were used to negative control.

**Table 3:** Classification of biofilm formation abilities by Mtp method

Cut-off value calculation	Mean of OD^a^ values results	Biofilm formation abilities
OD > 4×ODc^b^	OD > 1.826	Strong
2×ODc < OD ≤ 4×ODc	0.913 < OD ≤ 1.826	Moderate
ODc < OD ≤ 2×ODc	0.456 < OD ≤ 0.913	Weak
OD ≤ ODc	OD ≤ 0.456	None

^**a**^ Optical density

^**b**^ Optical density cut-off (ODc) = average OD of negative control + 3×standard deviation (SD) of negative control.

### ST typing, SCC*mec* typing and detection of biofilm-related genes

The MRSA strains in this study were derived from the strains studied by Wang B et al. [45]and Zhu H et al. [46]. , based on the results of whole genome sequencing(WGS) and bioinformatics analysis completed in the above studies, for example, STs were inferred using the *S. aureus* MLST database (https://pubmlst.org/organisms/staphylococcus aureus). SCC*mec*-types were predicted using the Center for Genomic Epidemiology website (https://cge.cbs.dtu.dk/services). Additionally, WGS data were used to identify biofilm-related genes using ABRicate v1.01 (https://github.com/tagann/abricate) using the VFDB database.

### Statistical analysis

Prism 6 software (GraphPad, La Jolla, CA, USA) was utilized for the analysis of experimental data. A significance level of *p* < 0.05 was considered statistically.

### Electronic supplementary material

Below is the link to the electronic supplementary material.

Supplementary Material 1

Supplementary Material 2

## Data Availability

The datasets generated during the current study are available from the corresponding author upon reasonable request. Most of the data is included in this published article.

## Declarations

### Reliability of experimental methods

All the experimental methods in this article were carried out in accordance with relevant guidelines and regulations.

## References

[1] Yamazaki Y (2024). The role of Staphylococcus aureus quorum sensing in cutaneous and systemic infections. Inflamm Regen.

[2] Rasigade JP, Dumitrescu O, Lina G (2014). New epidemiology of Staphylococcus aureus infections. Clin Microbiol Infect.

[3] Boles BR, Horswill AR (2011). Staphylococcal biofilm disassembly. Trends Microbiol.

[4] Song M (2024). Staphylococcus aureus and biofilms: transmission, threats, and promising strategies in animal husbandry. J Anim Sci Biotechnol.

[5] Herrmann M (1988). Fibronectin, fibrinogen, and laminin act as mediators of adherence of clinical staphylococcal isolates to foreign material. J Infect Dis.

[6] Franz S (2011). Immune responses to implants - a review of the implications for the design of immunomodulatory biomaterials. Biomaterials.

[7] McDevitt D (1994). Molecular characterization of the clumping factor (fibrinogen receptor) of Staphylococcus aureus. Mol Microbiol.

[8] Barbu EM (2014). SdrC induces staphylococcal biofilm formation through a homophilic interaction. Mol Microbiol.

[9] Cue D, Lei MG, Lee CY (2012). Genetic regulation of the intercellular adhesion locus in staphylococci. Front Cell Infect Microbiol.

[10] Moormeier DE, Bayles KW (2017). Staphylococcus aureus biofilm: a complex developmental organism. Mol Microbiol.

[11] Wang J (2021). Structural insights into the intermolecular interaction of the adhesin SdrC in the pathogenicity of Staphylococcus aureus. Acta Crystallogr F Struct Biol Commun.

[12] Herman-Bausier P (2015). Staphylococcus aureus Fibronectin-binding protein A mediates cell-cell adhesion through Low-Affinity Homophilic Bonds. mBio.

[13] Park PW (1996). Molecular cloning and expression of the gene for elastin-binding protein (ebpS) in Staphylococcus aureus. J Biol Chem.

[14] Downer R (2002). The elastin-binding protein of Staphylococcus aureus (EbpS) is expressed at the cell surface as an integral membrane protein and not as a cell wall-associated protein. J Biol Chem.

[15] Peng Q et al. A review of Biofilm formation of Staphylococcus aureus and its regulation mechanism. Antibiot (Basel), 2022. 12(1). 10.3390/antibiotics12010012 PMC9854888 36671212

[16] Verderosa AD, Totsika M, Fairfull-Smith KE (2019). Bacterial Biofilm Eradication Agents: Curr Rev Front Chem.

[17] Evans JJ, Bolz DD (2019). Regulation of virulence and antibiotic resistance in Gram-positive microbes in response to cell wall-active antibiotics. Curr Opin Infect Dis.

[18] Khatoon Z (2018). Bacterial biofilm formation on implantable devices and approaches to its treatment and prevention. Heliyon.

[19] Kaplan JB (2018). Extracellular polymeric substance (EPS)-degrading enzymes reduce staphylococcal surface attachment and biocide resistance on pig skin in vivo. PLoS ONE.

[20] O’Gara JP (2017). Into the storm: chasing the opportunistic pathogen Staphylococcus aureus from skin colonisation to life-threatening infections. Environ Microbiol.

[21] Watkins KE, Unnikrishnan M (2020). Evasion of host defenses by intracellular Staphylococcus aureus. Adv Appl Microbiol.

[22] Davies D (2003). Understanding biofilm resistance to antibacterial agents. Nat Rev Drug Discov.

[23] Indrawattana N (2013). Staphylococcus aureus clinical isolates: antibiotic susceptibility, molecular characteristics, and ability to form biofilm. Biomed Res Int.

[24] Craft KM (2019). Methicillin-resistant Staphylococcus aureus (MRSA): antibiotic-resistance and the biofilm phenotype. Medchemcomm.

[25] Archer NK (2011). Staphylococcus aureus biofilms: properties, regulation, and roles in human disease. Virulence.

[26] Chen Q (2020). Biofilm formation and prevalence of adhesion genes among Staphylococcus aureus isolates from different food sources. Microbiologyopen.

[27] Chen L et al. Biofilm Production ability, virulence and Antimicrobial Resistance genes in Staphylococcus aureus from various Veterinary hospitals. Pathogens, 2020. 9(4). 10.3390/pathogens9040264 PMC7238219 32260416

[28] Uribe-Garcia A (2021). Frequency and expression of genes involved in adhesion and biofilm formation in Staphylococcus aureus strains isolated from periodontal lesions. J Microbiol Immunol Infect.

[29] Patti JM (1995). Critical residues in the ligand-binding site of the Staphylococcus aureus collagen-binding adhesin (MSCRAMM). J Biol Chem.

[30] Pereyra EA (2016). Detection of Staphylococcus aureus adhesion and biofilm-producing genes and their expression during internalization in bovine mammary epithelial cells. Vet Microbiol.

[31] Khoramian B (2015). Comparison of virulence factors and biofilm formation among Staphylococcus aureus strains isolated from human and bovine infections. Microb Pathog.

[32] Roche FM (2004). The N-terminal A domain of fibronectin-binding proteins A and B promotes adhesion of Staphylococcus aureus to elastin. J Biol Chem.

[33] Atshan SS et al. *Prevalence of adhesion and regulation of biofilm-related genes in different clones of Staphylococcus aureus* J Biomed Biotechnol, 2012. 2012: p. 976972. 10.1155/2012/976972 PMC3372070 22701309

[34] Keinhorster D (2019). Function and regulation of Staphylococcus aureus wall teichoic acids and capsular polysaccharides. Int J Med Microbiol.

[35] Melles DC (2008). Serotyping of Dutch Staphylococcus aureus strains from carriage and infection. FEMS Immunol Med Microbiol.

[36] Rozemeijer W (2015). Evaluation of approaches to monitor Staphylococcus aureus virulence factor expression during human disease. PLoS ONE.

[37] Salimena AP (2016). Genotypic and phenotypic detection of capsular polysaccharide and biofilm formation in Staphylococcus aureus isolated from bovine milk collected from Brazilian dairy farms. Vet Res Commun.

[38] Seaman P (2004). Susceptibility of capsular Staphylococcus aureus strains to some antibiotics, triclosan and cationic biocides. J Antimicrob Chemother.

[39] Bowler PG (2018). Antibiotic resistance and biofilm tolerance: a combined threat in the treatment of chronic infections. J Wound Care.

[40] Costerton JW, Stewart PS, Greenberg EP (1999). Bacterial biofilms: a common cause of persistent infections. Science.

[41] Hall-Stoodley L, Costerton JW, Stoodley P (2004). Bacterial biofilms: from the natural environment to infectious diseases. Nat Rev Microbiol.

[42] Yang X (2017). Multiresistant ST59-SCCmec IV-t437 clone with strong biofilm-forming capacity was identified predominantly in MRSA isolated from Chinese children. BMC Infect Dis.

[43] Yu S (2020). Investigation of biofilm production and its association with genetic and phenotypic characteristics of OM (osteomyelitis) and non-OM orthopedic Staphylococcus aureus. Ann Clin Microbiol Antimicrob.

[44] Pinto RM (2019). Impact of nanosystems in Staphylococcus aureus biofilms treatment. FEMS Microbiol Rev.

[45] Wang B (2022). Methicillin-resistant Staphylococcus aureus in China: a multicentre longitudinal study and whole-genome sequencing. Emerg Microbes Infect.

[46] Zhu H (2022). Comparison of molecular characteristics between Methicillin-resistant and -susceptible Staphylococcus aureus Clinical isolates by whole-genome sequencing. Infect Drug Resist.

[47] Kranjec C, et al. A bacteriocin-based antimicrobial formulation to effectively disrupt the cell viability of methicillin-resistant Staphylococcus aureus (MRSA) biofilms. Volume 6. NPJ Biofilms Microbiomes; 2020. p. 58. 1. 10.1038/s41522-020-00166-4 PMC7710749 33268776

[48] Mirzaee M (2015). Relationship between adhesin genes and biofilm formation in Vancomycin-intermediate Staphylococcus aureus clinical isolates. Curr Microbiol.

