# Supplementary material for: Biofilm-producing ability of methicillin-resistant Staphylococcus aureus clinically isolated in China
Source: BMC Microbiol. 2024 Jul 3;24:241. doi: 10.1186/s12866-024-03380-8 (PMC11223284; doi:10.1186/s12866-024-03380-8)
Supplement: Supplementary file 1 — Supplementary Material 1 [file 12866_2024_3380_MOESM1_ESM.pdf]

Supplementary material 1

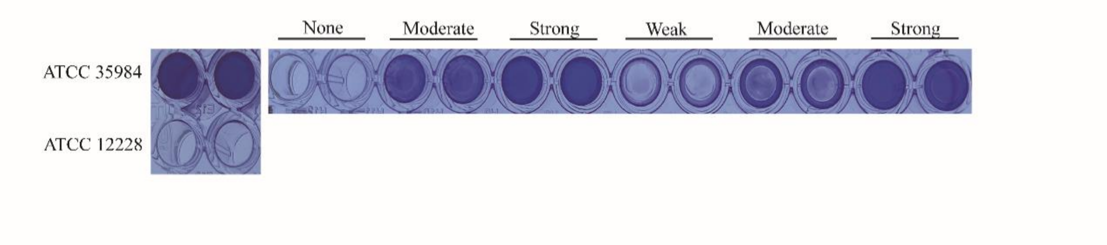

Biofilm semi-quantitative method to detect MRSA strains with strong, medium, weak or none biofilm formation ability in 96-well plates. ATCC35984 and ATCC12228 were used as biofilm positive and negative controls respectively.

Supplementary material 2

List of strains used in this study and their ST details.
